# Modeling Post-death Transmission of Ebola: Challenges for Inference and Opportunities for Control

**DOI:** 10.1038/srep08751

**Published:** 2015-03-04

**Authors:** Joshua S. Weitz, Jonathan Dushoff

**Affiliations:** 1 School of Biology, Georgia Institute of Technology, Atlanta, GA, USA; 2 School of Physics, Georgia Institute of Technology, Atlanta, GA, USA; 3 Department of Biology, McMaster University, Hamilton, ON, Canada; 4 Institute of Infectious Disease Research, McMaster University, Hamilton, ON, Canada

## Abstract

Multiple epidemiological models have been proposed to predict the spread of Ebola in West Africa. These models include consideration of counter-measures meant to slow and, eventually, stop the spread of the disease. Here, we examine one component of Ebola dynamics that is of ongoing concern – the transmission of Ebola from the dead to the living. We do so by applying the toolkit of mathematical epidemiology to analyze the consequences of post-death transmission. We show that underlying disease parameters cannot be inferred with confidence from early-stage incidence data (that is, they are not “identifiable”) because different parameter combinations can produce virtually the same epidemic trajectory. Despite this identifiability problem, we find robustly that inferences that don't account for post-death transmission tend to underestimate the basic reproductive number – thus, given the observed rate of epidemic growth, larger amounts of post-death transmission imply larger reproductive numbers. From a control perspective, we explain how improvements in reducing post-death transmission of Ebola may reduce the overall epidemic spread and scope substantially. Increased attention to the proportion of post-death transmission has the potential to aid both in projecting the course of the epidemic and in evaluating a portfolio of control strategies.

A recent, influential modeling paper concluded, based on data available as of September 2014, that the ongoing Ebola epidemic in Guinea, Liberia and Sierra Leone had the potential to exceed 1 million new cases by mid-January 2015, in the absence of intervention^1^. Even with intervention and changes in behavior, a follow-up study by an independent group in October 2014 estimated that 100, 000 additional cases could be expected in Liberia alone by mid-December 2014, unless a coordinated, large-scale response is implemented rapidly^2^. These predictions leveraged the structure of previous epidemiological models^3^,^4^ that encapsulate the infection cycle of Ebola virus disease (EVD), by tracking the dynamics and interactions of different types of individuals within a population including Susceptible, Exposed, Infectious and Removed types. Exposed individuals are infected but not yet infectious (i.e., also referred to as latently infected). In a SEIR model representation of EVD dynamics, the R class accounts for two types of individuals: those who recovered from the disease and those who have died from the disease (and are therefore “removed”).

However, a complication in modeling EVD arises because EVD may be contracted by direct contact with bodily uids from individuals who are alive and from those who have died from the disease^5^,^6^. In the early stages of the present epidemic, contact tracing of 701 individuals confirmed to have been infected with EVD in the ongoing epidemic found that 67 patients reported contacts with individuals who died of EVD, but not with any living EVD cases, while 148 patients reported contacts with both living and dead individuals infected with EVD^7^, consistent with approximately 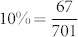 to 
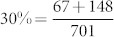
 of cases caused by post-death transmission. If funerals and burial rites can act as “super-spreader” events^8^,^9^,^10^, the true fraction may lie outside this range: for example, Legrand and colleagues^4^ estimated that 2/3 of the total 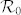 for the 1995 EVD outbreak in the Democratic Republic of Congo could be attributed to post-death transmission. The improvement of burial practice has become an issue of ongoing concern, e.g., Ref. 11. Some early^4^ and recent (e.g., Refs. 2, 10, 12, 13) epidemiological models of EVD have incorporated a D class, thereby distinguishing between recovered and dead individuals. Other models treat post-death transmission implicitly, by increasing the effective transmission rate and/or duration in the I class^1^,^14^. Yet, the implications of post-death transmission for inferences about epidemic spread have not been evaluated systematically. As we show, uncertainty in the relative force of infection before and after death has a number of consequences for estimating 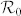 and the potential for control of the ongoing Ebola epidemic.

## Results

### The basic reproductive number, 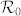, of EVD includes the effects of post-death transmission

The basic reproductive number, 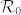, denotes the average number of secondary cases caused by a single infected individual in an otherwise susceptible population. The criterion for epidemic spread in standard epidemiological models is that 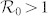 so that the initial infection gives rise, on average, to more than one infected case.

**Figure 1:**
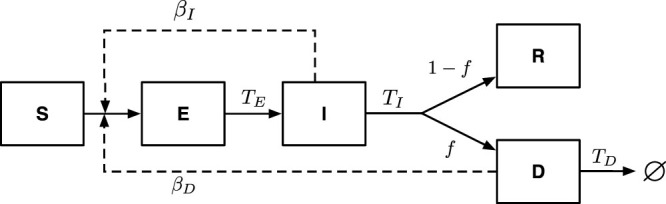
Schematic of the SEIRD model, i.e., the dynamics of Susceptible, Exposed (i.e., latently infected), Infectious, Recovered and Dead (but still infectious) individuals. Solid arrows denote transitions between states. Dashed arrows denote that transmission depends on interactions between *S* and *I* individuals or between *S* and *D* individuals. Parameters *β_I_* and *β_D_* are transmission rates, *T_E_*, *T_I_* and *T_D_* are the average periods in the *E*, *I* and *D* class, respectively, and *f* is the fraction of individuals who die of EVD.

In a conventional SEIR model, EVD transmission between infected and susceptible individuals occurs at an average rate *β_I_* over a period of infectiousness *T_I_*. A fraction *f* of infected individuals die and the remainder, 1 − *f*, recover and are assumed to be permanently immune to subsequent infection. A SEIRD model includes an additional transmission route: dead individuals can transmit EVD to susceptible individuals at a rate *β_D_* over a period of infectiousness *T_D_*, after which they are permanently removed from the system via burial or loss of infectiousness (see Figure 1). The methods provides the mathematical details of the model. The basic reproductive number in the SEIRD model is:
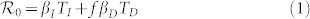
The first term denotes the average number of secondary infections due to contact with an infected individual before-death. The second term denotes the average number of secondary infectious due to contact with an infected individual after-death. The number of cases arising from contact with dead individuals is modulated by the fraction, *f*, of infected individuals that die due to EVD. In contrast, the basic reproductive number in the SEIR model is:
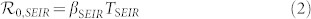
It might seem that the basic reproductive number of a SEIRD model should tautologically exceed that of a SEIR model. In fact, this will depend on how parameters are estimated. If the SEIR model is fit from data, then *β*_S*EIR*_ and *T*_S*EIR*_ will reflect transmission from both living and dead infectious individuals. Thus, we ask: What is the change in the estimated value of 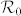 given alternative model frameworks meant to explain the same infected case data?

### Identifiability problems in estimating the basic reproductive number, 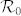


The SEIRD model, like the SEIR, SIR and other epidemiological models, predicts that there should be an exponential increase in the number of infected cases, i.e., *I*(*t*) ~ *e^λt^*, after an initial transient phase and before interventions, large-scale behavioral changes or population-level depletion of susceptibles have taken effect^16^. The exponential growth rate, *λ*, is a function of epidemiological parameters, including the transmission rate and 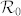
17,18. For EVD, prior information is available to constrain the mean duration of the latent phase on the order of 8–12 days^7^,^19^, the mean infectious period before death or recovery on the order of 5–9 days^1^,^7^ and the fraction of disease-induced mortality of approximately 70%^3^,^7^. However, even with these prior constraints, theory does not predict a one-to-one relationship between 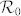 – the feature we want to infer – and *λ* – the feature that we can measure. This lack of a one-to-one relationship gives rise to a so-called identifiability problem in estimating epidemiological parameters, including 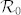, from early-stage epidemic data alone. The Supplementary Information presents a rationale for why identifiability problems arise more generally when fitting epidemiological models.

To examine the identifiability problem as it pertains to EVD, we fit both the SEIR and SEIRD models to an exponentially growing epidemic with rate 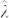 for which the number of cases is increasing with a characteristic time of 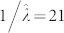 days. Further, we assume that *T_E_* = 11 days, *T_I_* = 6 days and *f* = 0.7. We utilize standard epidemiological methods to infer 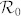 for the SEIR model and, in turn, *β*_S*EIR*_, given observations of 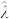 (see Eq. 10). We find a point estimate of *β*_S*EIR*_ of 0.33 and a corresponding 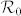 for the SEIR model of 1.95. Uncertainty in the duration of periods, risk of mortality and noise in epidemic case count data would lead to corresponding uncertainty in the value of 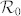.

In contrast, there are three unknown parameters in the SEIRD model: *β_I_*, *β_D_* and *T_D_*. The time to burial is, in part, culturally determined, with prior estimates of 2 days applied to Ebola outbreaks in Uganda and the Democratic Republic of Congo^4^ having been carried forward to current models (e.g. Ref. 10). Yet, given the size of the outbreak, additional delays between death and burial are likely. Even with a fixed value of *T_D_*, the transmission rates *β_I_* and *β_D_* remain unknown. Hence, trying to fit a SEIRD model to epidemic case data poses an identifiability problem. That is to say: there are potentially many combinations of transmission parameters, *β_I_* and *β_D_* that can yield the same observed epidemic growth rate. Here, we consider three scenarios, where *T_D_* = 2, 4 and 6 days. For each scenario, we must solve for the combination of *β_I_* and *β_D_* that yield the epidemic growth rate *λ* = 1/21. The mathematical details are in the Supplementary Information.

**Figure 2:**
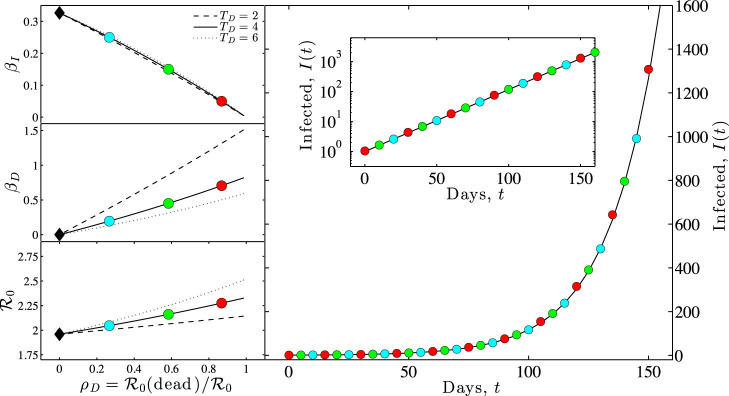
Identifiability problems in inferring epidemiological parameters from early-stage epidemic growth data. (Model) The dynamics are that of an SEIR model (diamond) and SEIRD model (lines and circles) with epidemiological parameters *T_E_* = 11, *T_I_* = 6 and *f* = 0.7. For the SEIRD model, three scenarios are considered where *T_D_* = 2, 4 and 6 days. For each scenario, we find a continuum of combinations of *β_D_* and *β_I_* that lead to the same early-stage epidemic growth rate, *λ* = 1/21. (Left) The calculated values of *β_I_*, *β_D_* and 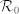 (from top-to-bottom) as a function of 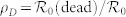. The focal parameter combinations for the scenario *T_D_* = 4 are, to two significant digits, [*ρ_D_* = 0.27, *β_I_* = 0.25, *β_D_* = 0.20 and 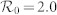], [*ρ_D_* = 0.58, *β_I_* = 0.15, *β_D_* = 0.45 and 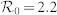] and [*ρ_D_* = 0.87, *β_I_* = 0.05, *β_D_* = 0.71 and 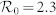], for cyan, green and red circles respectively. (Right panel) Simulated dynamics of infectious case counts for the three focal parameter combinations at intervally spaced time-points. The solid line denotes the expected increase in *I*(*t*) as predicted from theory. *Key point: Varying degrees of post-death transmission can yield the same characteristic epidemic-growth curves.*

For a given value of *T_D_*, we evaluate a continuum of models in which the proportion of 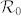 attributable to post-death transmission varies from 0 to 1. We define this fraction as 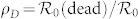. We find a negative relationship between the estimated pre- and post-death transmission rate (compare Figure 2-upper left and middle-left panels). This negative relationship is a consequence of trying to fit the same observed case data while modifying the relative importance of pre- and post-death transmission. Importantly, the point-estimate of 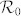 increases with increasing force of transmission post-death (Figure 2-lower left). Increasing post-death transmission implies that the average infectious period also increases. As a consequence, there are fewer epidemic generations that nonetheless led to the same rise in cases. This means that the average number of secondary infections per infected individual must be higher. This is a generic feature of epidemiological models. Here, the predicted growth rate for epidemics with these distinct epidemiological parameters are equivalent − *λ* = 1/21 (Figure 2-right) – despite the differences in underlying rates. We provide further support for this result by investigating dynamics in which the period of infectiousness of individuals who recover from EVD exceeds that of the infectious period for individuals who die from EVD (see the Supplementary Information). Even in this scenario, increases in the relative fraction of post-death transmission are associated with increases in the inferred value of 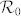 given the same epidemic case data.

### Challenges in fitting early-stage epidemic data of EVD in West Africa due to identifiability problems

**Figure 3:**
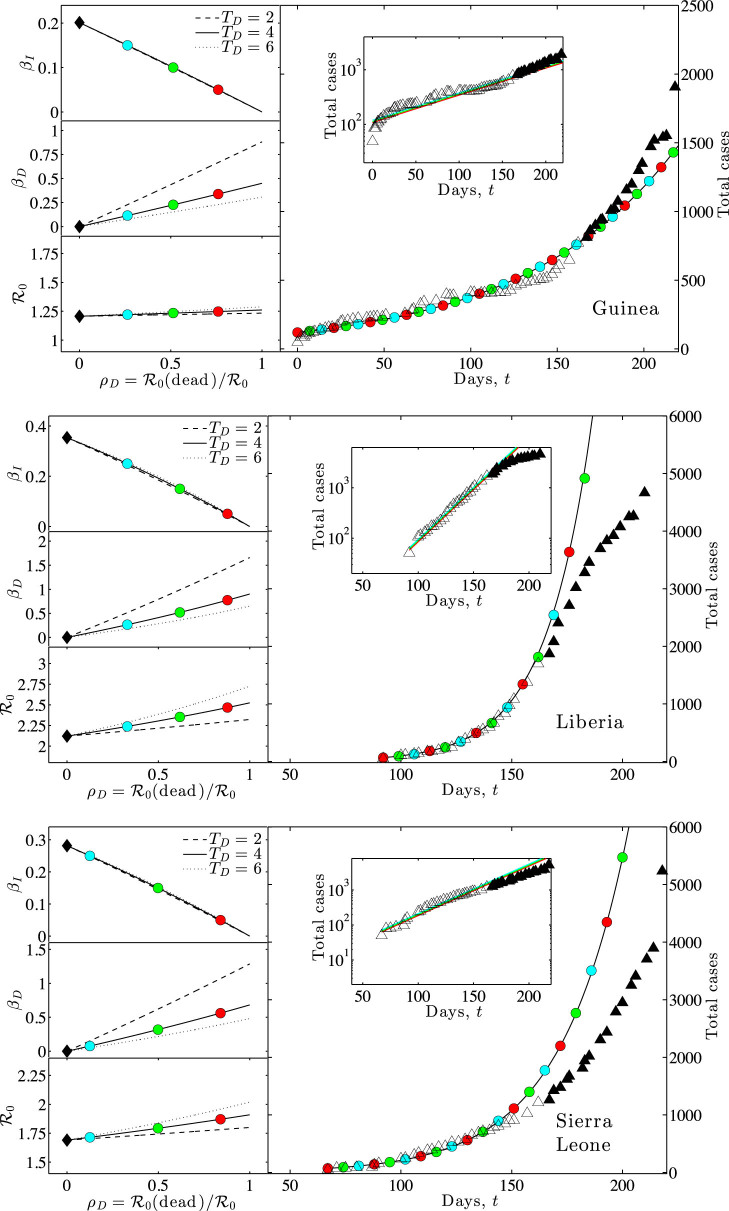
Inferring epidemiological parameters from early-stage Ebola epidemic case data in West Africa. (Data) A portion of cumulative case data^15^ was used to identify an exponential growth rate 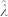 (see Methods). The calibration regime for model fits is denoted in open triangles, where Day 50 is 5/11/14, Day 100 is 6/30/14, Day 150 is 8/19/14 and Day 200 is 10/8/14. (Model) The dynamics are that of an SEIR model (diamond) and SEIRD model (lines and circles) with epidemiological parameters *T_E_* = 11 (modeled as a gamma distribution), *T_I_* = 6 and *f* = 0.7. For the SEIRD model, three scenarios are considered where *T_D_* = 2, 4 and 6 days. (Left) The calculated values of *β_I_*, *β_D_* and 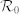 (from top-to-bottom) as a function of 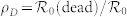. (Right panel) Dynamics of epidemics for the three focal parameter combinations (colored circles). For each country, all three focal parameter combinations lead to the same country-specific exponential epidemic growth rate, 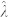.

The identifiability problem, described in the previous section, suggests why it is more difficult than has been recognized to ascertain the mechanistic details of EVD transmission from early-stage epidemic data alone. Here, we investigate case data from three countries: Guinea, Liberia and Sierra Leone (data from Ref. 15; see Table 1 for more information). We use an exponential growth curve fit to the cumulative case counts as a target to investigate multiple possible scenarios (Figure 3). For this fit, we extend our SEIRD model to include a more realistic distribution period for the E class^7^,^19^. The exposed (i.e, latently infected) period is modeled as a gamma distribution with mean of 11 days and 6 classes, so that the standard deviation is 4.5 days (see Supplementary Information for more details). We use the generating-function approach of Wallinga and Lipsitch^18^ (see the Supplementary Information) to estimate 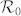 from *λ* while accounting for the chosen time distributions within the E, I and D classes. For each country, the resulting model predictions have two key features (see Figure 3). First, multiple scenarios with varying ratios of transmission risk from living and dead individuals all fit the data equally well. Second, estimates that neglect post-death transmission tend to under-estimate 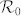. The bottom-left panels of Figure 3 all show an increase in 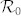 that varies with the fraction of cases caused by post-death transmission, *ρ_D_*. The increase in 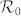 due to post-death transmission is of concern. However, there is a tradeoff: larger *ρ_D_* means not only a larger 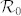, but also a larger potential impact of reducing post-death transmission.

### Reduction in transmission risk after death can have substantial epidemiological benefits

**Figure 4:**
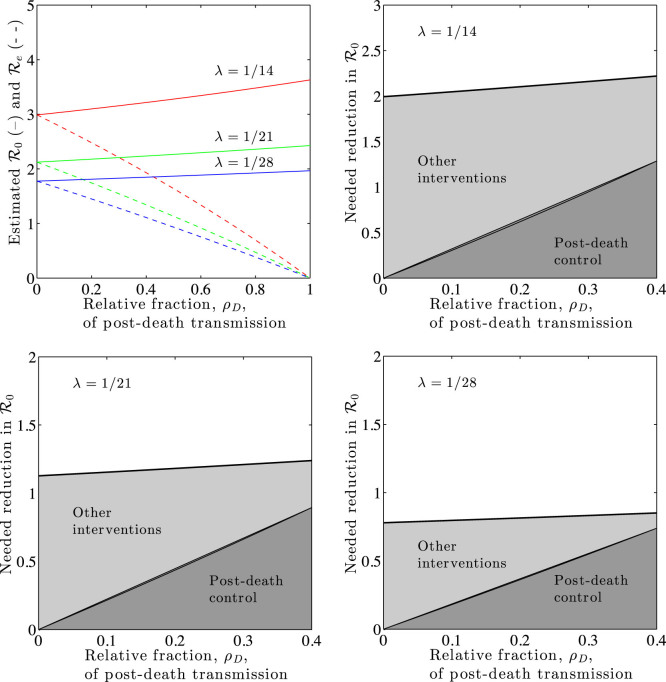
The effect of controlling post-death transmission of EVD outbreaks with different epidemic growth rates *λ*. (Topleft) Basic reproductive numbers, 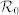, without intervention (solid lines) compared to effective reproductive numbers, 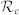, by eliminating post-death transmission (dashed lines). Reproductive numbers are plotted against the fraction *ρ_D_* of secondary infections due to dead-to-living transmission. For all scenarios, 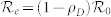. (Other panels) Break-down of the needed reduction in 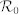 to reach a value of 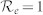 for each of the characteristic epidemic growth rates examined in the top-left panel. The dark-shaded region denotes the reduction in secondary cases due to elimination of post-death transmission as a function of *ρ_D_*. The light-shaded region denotes the additional reduction in secondary cases necessary if post-death transmission is eliminated.

We evaluate the benefits of control in a SEIRD representation of EVD using a gamma distributed E class period. Three scenarios are considered, in which the characteristic epidemic growth times are 1/*λ* = 14, 21 and 28 days and for which we assume *T_D_* = 3 days. Figure 4 summarizes our central findings. We find, as before, that 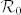 is an increasing function of *ρ_D_*, the proportion of transmission that occurs post-death. We also find that the inferred basic reproductive number increases with increasing epidemic growth rates. These estimates can be used to evaluate the benefit of control strategies that eliminate (even partially) post-death transmission. In the limit that all post-death transmission is eliminated, the effective reproductive number would be 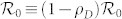. In this limit, 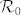 is reduced by 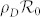, a substantial amount given estimates of *ρ_D_* in the range of 10%–30%^7^. For example, in the scenarios evaluated, control of post-death transmission reduces 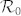 by ≈0.2–1 secondary transmission per infected individual. Thus, controlling post-death transmission of EVD could be an important component of epidemic control.

## Discussion

The relative importance of post-death transmission is difficult to estimate from epidemic growth rate data alone, and has important implications for estimates of key epidemiological quantities, and for prediction. This difficulty is due to what is classically termed an “identifiability problem” - relevant to EVD and to other emerging or poorly characterized infectious diseases. Improved estimation of the relative proportion of post-death transmission would mitigate this problem and improve reliability in estimates of 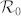. Despite the challenge in identifying epidemiological parameters, we robustly conclude that neglecting post-death transmission while fitting to epidemic growth rate tends to lead to underestimates of 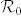. The degree of under-estimation depends on the fraction of post-death transmission which, in turn, depends on transmission risk and time spent in the community, in hospitals, and in burial ceremonies. Such underestimates are a potential concern for ongoing efforts to develop realistic models of Ebola dynamics and its control.

Here, we have focused on one feature of such control: the use of burial teams and other practices intended to reduce post-death transmission^11^. Burial teams are part of a diverse set of responses required to stop the spread of EVD^10^. These responses include behavioral changes, hospital interventions^1^,^20^, and (potentially) vaccination. More burial teams were needed, but recruiting was initially difficult due to stigmatization, lack of personal protective equipment, and insufficient compensation of workers^21^. Improvements in burial practices have been documented and are believed to have contributed to decreases in onwards transmission of EVD in the months after the case data analyzed here^11^. Previous reports have suggested that some individuals in the population experiencing the epidemic may have acquired immunity to Ebola as a result of sub-clinical infections^22^,^23^. If these individuals exist, and can be identified, they may be valuable contributors to response efforts if they can be recruited as family health-care workers^23^ or as part of burial teams. Moving forward, it is essential to consider the logistics of deploying burial teams efficiently and safely while balancing public health benefits and community norms^11^,^24^,^25^. A better understanding of post-death transmission can help to understand the EVD epidemic in West Africa and plan control efforts, hopefully leading in the long-term to control and elimination of the current outbreak.

## Methods

### SEIRD model of Ebola dynamics

The SEIRD model includes the dynamics of susceptible, exposed and infectious individuals, just as in the SEIR model. It differs in that the R class stands for recovered individuals while the D class stands for dead individuals, who are nonetheless infectious. The dynamics can be written as:
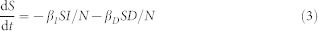

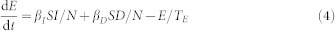

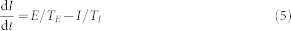

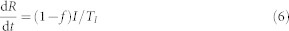

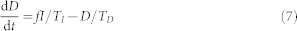
where *β_I_* is the transmission rate for contacts with infected individuals and *β_D_* is the transmission rate associated with contacts with dead individuals (who are nonetheless infectious). The other parameters are: *T_E_* the average exposed period, *T_I_* the average infectious period, *T_D_* the average period of infectiousness after death and *f* the fraction of infected individuals who die. In this model *N* = *S* + *E* + *I* + *R*. This model neglects birth/immigration of individuals and natural death/emigration. The conventional derivation of the transmission rate is that a susceptible individual interacts with a certain fixed number of individuals per unit time *m*, of which a fraction *I*/*N* are infectious, and only a fraction *p* of which lead to transmission. The transmission rate *β_I_* is a product of *m* and *p*. Similarly, here we assume that dead individuals are contacted by a certain fixed number of individuals per unit time *n*, of which a fraction *S*/*N* are susceptible, and only a fraction *q* of contacts lead to transmission. The transmission rate *β_D_* is a product of *n* and *q*. The SEIRD model can be extended further to take into account the possibility that the duration of the exposed infectious and dead periods are non-exponential.

### Estimating the basic reproductive number, 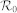, for the SEIR and SEIRD models given exponential intra-class period distributions

Infected case data can then be used to estimate unknown epidemiological parameters, including the transmission rate and 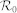. In the case of the SEIR model, the predicted exponential growth rate, *λ*, can be derived from a solution of the linearized dynamics near the value of (*S* = *N*, *E* = 0, *I* = 0, *R* = 0). The growth rate *λ* correspond to the largest eigenvalue of the Jacobian:
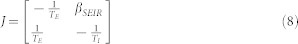
It can be shown that the growth of the number of infected cases is an exponential of the form *I*(*t*) = *I*_0_*e^λt^* where
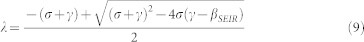
where *σ* ≡ 1/*T_E_* and *γ* ≡ 1/*T_I_*. The best-fit value of *β_SEIR_* can be inferred given a measured value 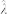 and prior estimates for *σ* and *γ*. For example,
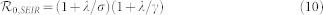
where 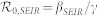, such that
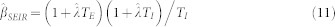


Similarly, given the SEIRD model, the predicted exponential growth rate *λ* of the number of infected cases corresponds to the largest eigenvalue of the linearized system near (*N*, 0, 0, 0, 0), of which only the variables *E*(*t*), *I*(*t*) and *D*(*t*) must be tracked. The Jacobian of this subsystem is:
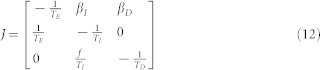
The solutions can be computed exactly.

### Case data information

Cumulative case count data from Guinea, Liberia and Sierra Leone was used as the target of model fits in Figure 3. These data sets were downloaded from Caitlin Rivers' publicly available github site^15^. The time periods over which data is calibrated is shown in Table 1. The start date was selected based on the first day at which the cumulative case count exceeded 50. The final date was set at the end of August, coinciding with reported increases in intervention and widespread dissemination of the severity of the outbreak^10^. The exponential fits are based on log-transformed cumulative case counts. Additional challenges for inference arise, in part, due to under-reporting^1^ and lags between incidence and reporting events^7^.

## Author Contributions

J.S.W. conceived of the project, developed analytic tools, performed analysis, analyzed data and wrote the manuscript. J.D. developed analytic tools, analyzed data, and edited the manuscript.

## Supplementary Material

Supplementary Information Supplementary Information

## Figures and Tables

**Table 1:** Data sources for model fits of SEIRD to Ebola epidemic data. The values of *T*_0_ and *T*_1_ denote the start and stop dates for the cumulative case data used for estimating the epidemic growth rate, 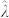. Estimates of the epidemic growth rate were based on linear regression of log-transformed cumulative case counts. The doubling time of the epidemic is defined as 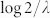

Country	*T*_0_	*T*_1_	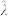	Doubling time
Guinea	3/22/14	8/31/14	0.011	61 days
Liberia	6/22/14	8/31/14	0.048	14 days
Sierra Leone	5/28/14	8/31/14	0.032	21 days
