# Supplementary material for: Modeling Post-death Transmission of Ebola: Challenges for Inference and Opportunities for Control
Source: Sci Rep. 2015 Mar 4;5:8751. doi: 10.1038/srep08751 (PMC4348651; doi:10.1038/srep08751)
Supplement: Supplementary Information [file srep08751-s1.pdf]

Supplementary Information:
Modeling Post-death Transmission of Ebola: Challenges for Inference and Opportunities for Control

Joshua S. Weitz[1,2,*] and Jonathan Dushoff[3,4]

[1] *School of Biology, Georgia Institute of Technology, Atlanta, GA, USA*
[2] *School of Physics, Georgia Institute of Technology, Atlanta, GA, USA*
[3] *Department of Biology, McMaster University, Hamilton, ON, Canada*
[4] *Institute of Infectious Disease Research, McMaster University, Hamilton, ON, Canada*

(Dated: February 3, 2015)

The roots of identifiability problems in estimating the basic reproductive number from early-stage epidemic growth data

The identifiability problem raised in the main text is a generic issue in epidemiology. By means of illustration, consider the spread of a disease that has no exposed stage, such that it can be suitably described using a SIR model. Further assume that the basic reproductive number of the disease is to be estimated from epidemic case data in which the number of cases is growing at a rate of $\hat{\lambda} = 1/28$. The basic reproductive number for a SIR model is βT_I, i.e., the transmission rate multiplied by the infectious period. The epidemic growth rate for a SIR model is $\lambda = \beta - 1/T_I$, i.e., the difference between the transmission and recovery rate. This can be written as: $\lambda = T_I(\mathcal{R}_0 - 1)$. Hence, consider three scenarios, in which the true infectious period is $T_I = 14$, 28 and 42 days. Each of these scenarios is compatible with the same epidemic growth rate $\hat{\lambda}$ given $\mathcal{R}_0 = 1.5$, 2.0 and 2.5. Figure S.1 illustrates this point using synthetic data. Note that for a given epidemic growth rate, diseases whose period of infectious is longer have larger basic reproductive numbers. In the example above, a disease with an infectious period of 14 days requires 2 infection cycles (on average) to increase in case count by a factor of e (2.718). Whereas, a disease with an infectious period of 28 days requires 1 infection cycle (on average) to increase in case count by a factor of e (2.718). This is the intuition behind the seemingly paradoxical result that diseases with longer infectious periods are estimated to have higher values of $\mathcal{R}_0$ when estimated via the same epidemic growth rate. Moreover, although the disease dynamics may appear indistinguishable at early stages of an epidemic, the long-term dynamics can be quite different. For example, Figure S.1 shows how diseases with higher values of $\mathcal{R}_0$ infect more people over the long-term despite having the same early time dynamics. Controlling a disease with a higher value of $\mathcal{R}_0$ is also more difficult.

Estimating the basic reproductive number, $\mathcal{R}_0$, for the SEIRD model given arbitrary intra-class period distributions

Wallinga and Lipsitch [1] established a formal connection between $\mathcal{R}_0$ and the epidemic growth rate, here: λ, such that

$$\mathcal{R}_0 = \frac{1}{M(-\lambda)} \tag{S.1}$$

where

$$M(z) = \int_0^\infty e^{za} g(a) \mathrm{d}a \tag{S.2}$$

The moment generating function $M(z)$ operates on the distribution $g(a)$ which, in epidemiological terms, is the normalized fraction of all secondary cases caused by an infectious individual at "age" a since infection. For example, if individuals are only infectious at a single age a_c after infection, then $g(a) = \delta(a - a_c)$ where $\delta(x)$ is the delta function. Similarly, if individuals recover from being infected at a rate γ, then $g(a) = \gamma e^{-\gamma a}$, i.e., an exponential distribution.

---

*Electronic address: jsweitz@gatech.edu; URL: http://ecotheory.biology.gatech.edu

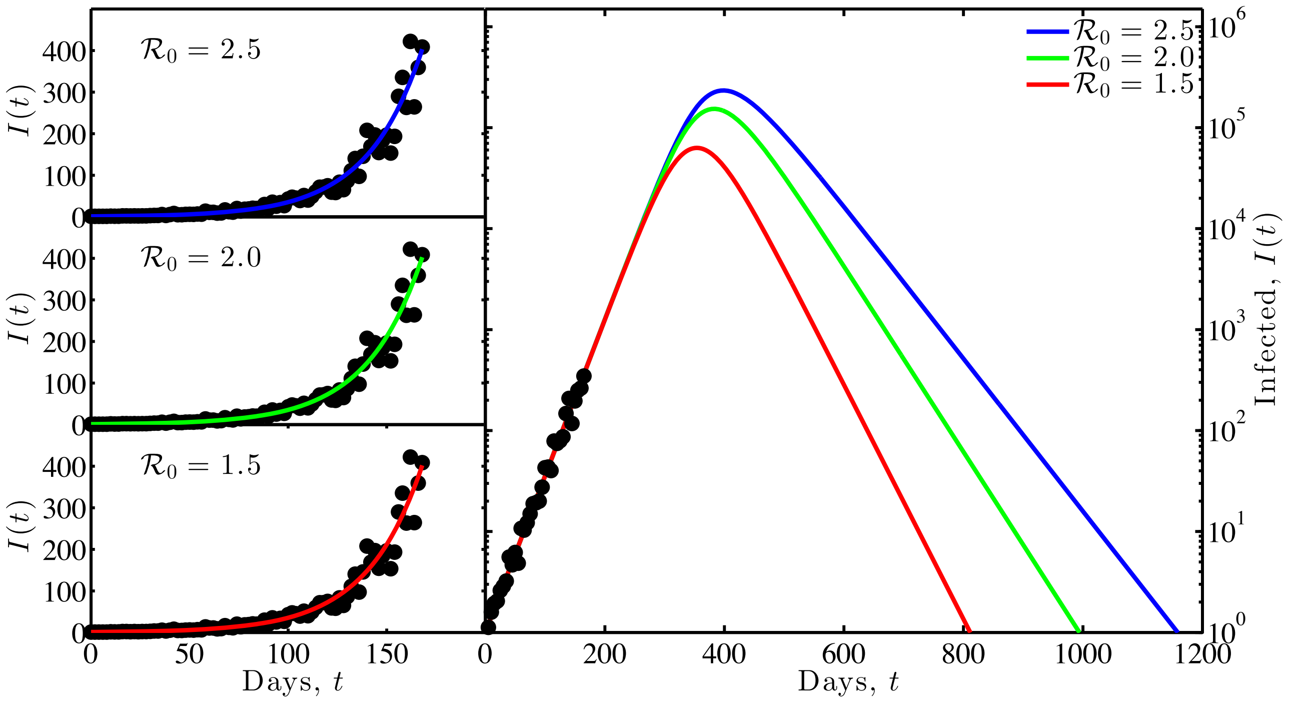

FIG. S.1: Identifiability problem in estimating $\mathcal{R}_0$ for a SIR model from exponential epidemic growth data. The synthetic data (black circles) is $I(t) \propto e^{\lambda t} e^{1+\psi}$ where $\lambda = 1/28$, corresponding to a characteristic time of 4 weeks and where ψ is a normally distributed random variable with mean 0 and standard deviation 0.2. The model fits correspond to solutions of SIR models in which $\beta = 0.107$, 0.0714, and 0.0595 days^{-1} and $T_I = 14$, 28 and 42 days respectively. The basic reproductive number in each case is $\mathcal{R}_0 = \beta T_I = 1.5$, 2.0 and 2.5 respectively. (Left panel) Each of the SIR model predictions fits the data equally well at early times, despite having very different basic reproductive numbers. (Right panel) The predictions of the long-term dynamics differ, with epidemic size increasing as a function of $\mathcal{R}_0$.

The advantage of this approach is that it is possible to uniquely identify the value of $\mathcal{R}_0$ given a measured epidemic growth rate $\hat{\lambda}$ and additional information on the age distributions for secondary infections.

For the SEIRD model, the appropriate generating function is:

$$M(z) = (1 - \rho_D) M_E(z) M_I(z) + \rho_D M_E(z) M_I(z) M_D(z) \tag{S.3}$$

where ρ_D is the fraction of secondary transmission due to post-death transmission and $1 - \rho_D$ is the fraction of secondary transmission due to pre-death transmission. We consider a gamma distributed exposed period with $T_E = 11$ days, and shape parameters $n_E = 6$ and $b_E = n_E/T_E$ (see Figure S.2) whose generating function is:

$$M_E(-\lambda) = \left(\frac{b_E}{b_E + \lambda} \right)^{n_E} \tag{S.4}$$

We consider here exponentially-distributed periods for the I and D classes. The generating functions are:

$$M_I(-\lambda) = \frac{\gamma}{\gamma + \lambda} \tag{S.5}$$

$$M_D(-\lambda) = \frac{\chi}{\chi + \lambda} \tag{S.6}$$

where $\gamma = 1/T_I$ and $\chi = 1/T_D$. Therefore for the SEIRD model, it is possible to estimate $\mathcal{R}_0$ using the generating function method given observations of an epidemic growth rate and suitable information on epidemiological modes and parameters.

This analysis assumed that the I and D classes are exponentially distributed with characteristic times of 6 and 3 days, respectively. A similar analysis can be performed in which the force of transmission is concentrated with different distributions, e.g., uniform, unimodal or even concentrated at the very end of a fixed epidemic period (so-called delta distributed).

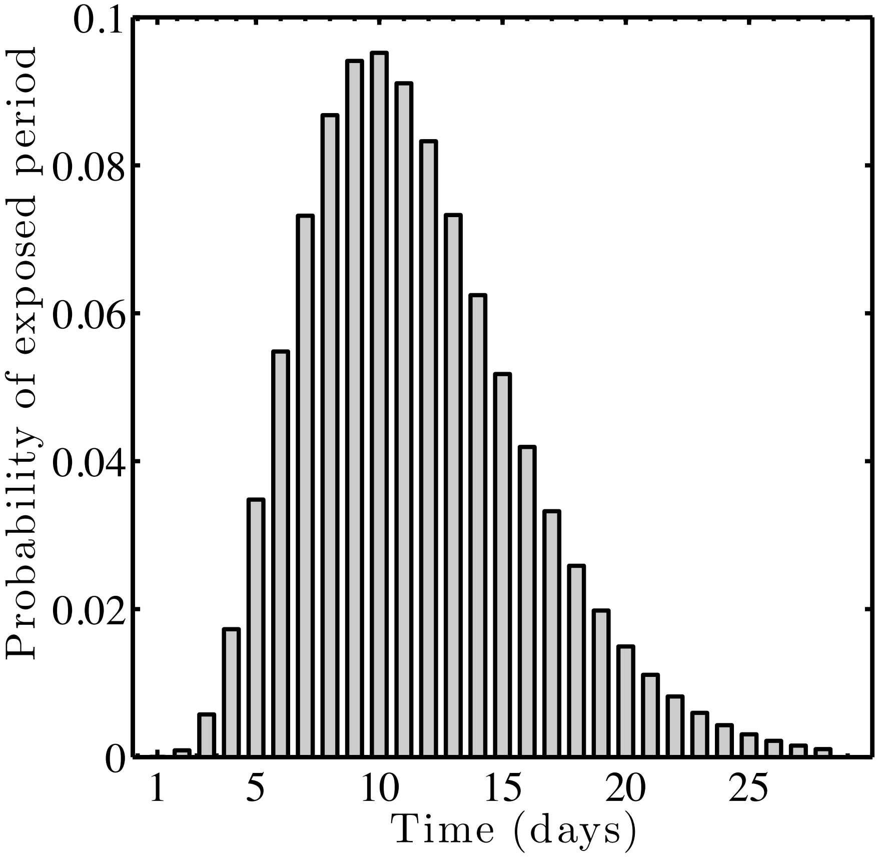

FIG. S.2: Gamma-distributed exposed period. The distribution has an average exposed period with $T_E = 11$ days, such that the shape parameters are $n_E = 6$ and $b_E = n_E/T_E$.

Effect of long recovery periods on the under-estimation of $\mathcal{R}_0$ due to post-death transmission

It is possible that the period of infectiousness differs among individuals who eventually die from EVD and those who do not. A WHO led study reported recovery times after hospitalization of approximately 16 days – much longer than the time to death of approximately 7 days [2]. It is not yet known how the transmission rates of infected individuals varies before recovery nor how transmission rates vary between individuals who recover vs. those who eventually die. Nonetheless, we investigate the potential effect of differences in infectious period on our conclusions, and show that our results are robust to this effect.

We utilize the generating function framework to estimate $\mathcal{R}_0$ as a function of ρ_D. Consider a SEIRD model in which individuals who die have an infectious period of T_{ID} and those who survive have an infectious period of T_{IR}. In that case:

$$\mathcal{R}_0 = \frac{1}{M(-\lambda)} \tag{S.7}$$

where λ is the epidemic growth rate and

$$M(z) = (1-\rho_D)\left((1-f)M_E(z)M_{IR}(z) + fM_E(z)M_{ID}(z)\right) + \rho_D M_E(z)M_{ID}(z)M_D(z). \tag{S.8}$$

In this generating function approach, the two new functions are:

$$M_{IR}(-\lambda) = \frac{\gamma_{IR}}{\gamma_{IR}+\lambda} \tag{S.9}$$

$$M_{ID}(-\lambda) = \frac{\gamma_{ID}}{\gamma_{ID}+\lambda} \tag{S.10}$$

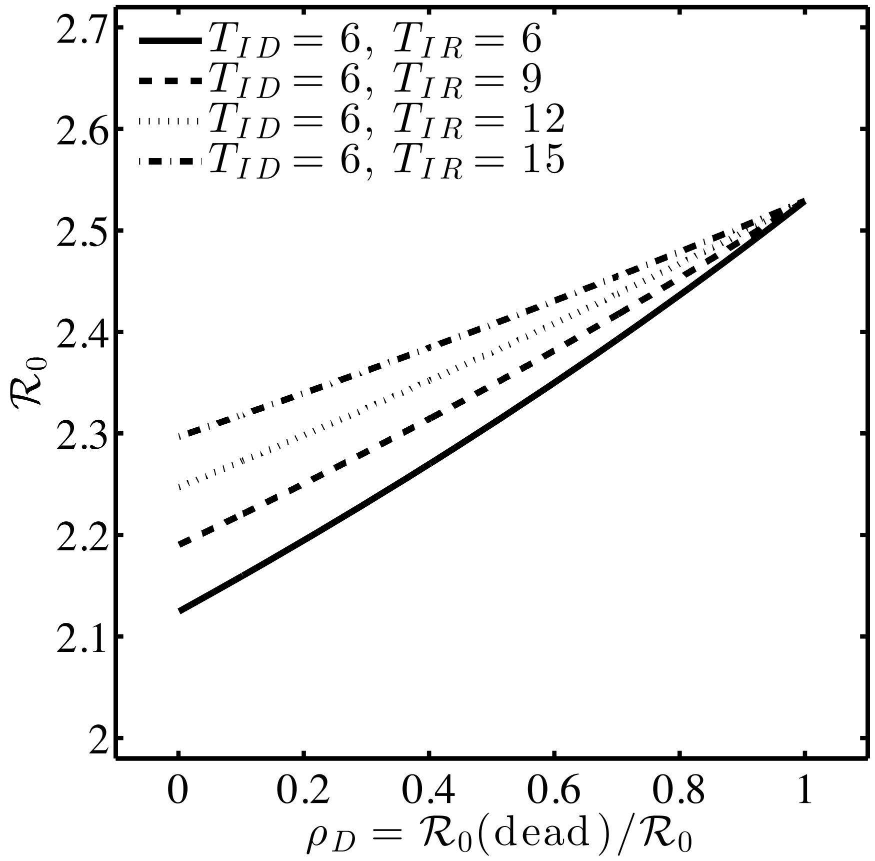

FIG. S.3: Effect of extended infectious period for individuals who recover on the under-estimation of $\mathcal{R}_0$ given post-death transmission. We utilize a generating function approach as described in the text given a gamma distributed exposed class with parameters as in Figure S.2 and other parameters: $f = 0.7$, $\lambda = 1/21$, $T_D = 4$, $T_{ID} = 6$. We consider the scenarios of $T_{IR} = 6, 9, 12$ and 15. Time is in units of days. The results are consistent with the previous analyses, i.e., $\mathcal{R}_0$ increases with ρ_D.

where $\gamma_{IR} = 1/T_{IR}$ and $\gamma_{ID} = 1/T_{ID}$. We conduct our analysis while modifying the values of T_{IR} to be 6, 9, 12 and 15 days while keeping T_{ID} constant at 6 days. We find that neglecting post-death transmission (i.e., $\rho_D = 0$) in all cases under-estimates $\mathcal{R}_0$ (see Figure S.3). The extent of this under-estimation problem is diminished if infectious individuals who eventually recover are infectious (at a constant rate) for longer. The baseline estimate of $\mathcal{R}_0$ increases in all cases with increasing T_{IR}, as expected for processes that extend the time to transmission given the same case data. Inferring the relative change in transmission in the course of recovering from EVD represents an important direction for future research.

---

1 Wallinga, J. & Lipsitch, M. How generation intervals shape the relationship between growth rates and reproductive numbers. *Proceedings of the Royal Society B: Biological Sciences* **274**, 599–604 (2007).

2 WHO Ebola Response Team. Ebola virus disease in West Africa – the first 9 months of the epidemic and forward projections. *New England Journal of Medicine* **371**, 1481–1495 (2014).
